# Supplementary material for: The Pathopharmacological Interplay between Vanadium and Iron in Parkinson’s Disease Models
Source: Int J Mol Sci. 2020 Sep 14;21(18):6719. doi: 10.3390/ijms21186719 (PMC7554808; doi:10.3390/ijms21186719)
Supplement: Supplementary file 1 [file ijms-21-06719-s001.zip › ijms-915387-supplementary.docx]

**Figure S1.** Cell culture time course: Dose response to vanadium on oxidative stress (mitochondrial viability) in undifferentiated, differentiating and differentiated CAD cells. Results from mitochondrial viability investigation showed that undifferentiated neuronal cells (**A**) are more sensitive to vanadium than differentiating (**B**) and differentiated cells (**C**), respectively, which correlates with the significantly higher levels of intracellular iron in undifferentiated (immature) versus differentiated cells (mature). Values are mean ± SD for *n* = 4 replicates.


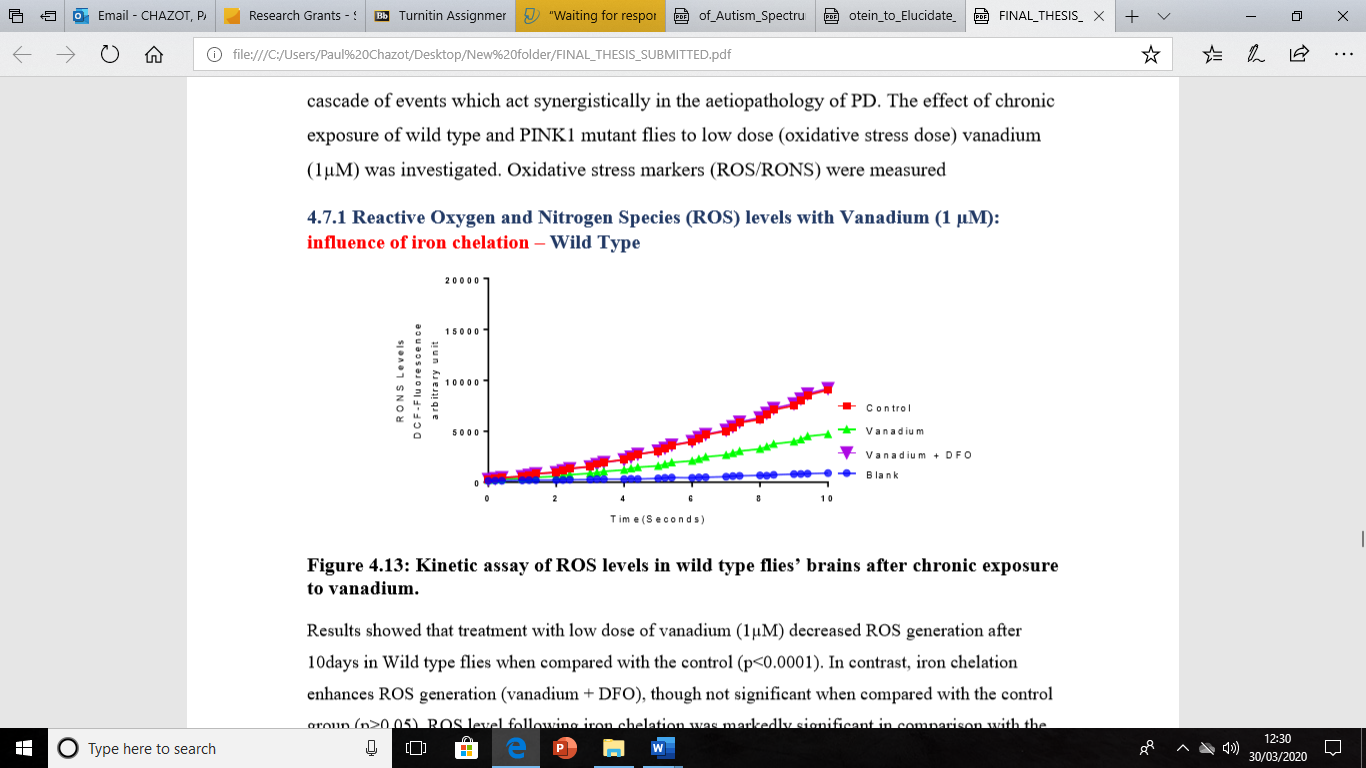


**Figure S2.** The influence of iron chelation following chronic exposure of WT Drosophila melanogaster low dose vanadium upon ROS/RONS production in WT flies brains on day 14. This figure showed that treatment with low dose of vanadium significantly reduced ROS/RONS generation after 14 days in WT flies, when compared with the control group. In contrast, iron chelation (DFO) completely reverses vanadium reduction of ROS/RONS in WT flies, which is statistically significant (****p<0.0001) when compared with vanadium only. All values are means ± SD, from 5 separate experiments, n=10 flies for each individual experiment; data were analysed with a repeated measure one-way ANOVA (with Tukey’s Multiple Comparisons Test).
